# Knowledge and Instance Mapping: architecture for premeditated interoperability of disparate data for materials

**DOI:** 10.1038/s41597-024-03006-8

**Published:** 2024-02-06

**Authors:** Jaleesia D. Amos, Zhao Zhang, Yuan Tian, Gregory V. Lowry, Mark R. Wiesner, Christine Ogilvie Hendren

**Affiliations:** 1 Center for the Environmental Implications of Nano Technology (CEINT), Durham, USA; 2 Civil & Environmental Engineering, Duke University, Durham, North Carolina 2770y8 USA; 3 Civil & Environmental Engineering, Carnegie Mellon University, Pittsburgh, Pennsylvania 15213 USA; 4 Department of Geological and Environmental Sciences, Appalachian State University, Boone, North Carolina 28608 USA; 5 Present Address: Lucideon M+P, Morrisville, North Carolina, 27560 USA

**Keywords:** Databases, Other nanotechnology

## Abstract

Predicting and elucidating the impacts of materials on human health and the environment is an unending task that has taken on special significance in the context of nanomaterials research over the last two decades. The properties of materials in environmental and physiological media are dynamic, reflecting the complex interactions between materials and these media. This dynamic behavior requires special consideration in the design of databases and data curation that allow for subsequent comparability and interrogation of the data from potentially diverse sources. We present two data processing methods that can be integrated into the experimental process to encourage pre-mediated interoperability of disparate material data: Knowledge Mapping and Instance Mapping. Originally developed as a framework for the NanoInformatics Knowledge Commons (NIKC) database, this architecture and associated methods can be used independently of the NIKC and applied across multiple subfields of nanotechnology and material science.

## Introduction

From the earliest moments in the scholarly examination of the implications of engineered nanomaterials on the environment, health and safety (nanoEHS), researchers understood that the material properties and model parameters used to predict the behavior and risks of this new class of materials would have to be different than those understood for bulk chemicals^1–4^. Where a chemical risk model for bulk materials might forecast exposure and hazard potential based on molecular weight and water solubility, the same novel functionalities driving the exciting potential of nanoscale materials – higher reactivity, or changed optical properties for example – made it clear that different parameters would be needed to accurately predict both their performance and their possible impacts. NanoEHS risk forecasting efforts hypothesized that for example specific surface area would be a better correlate than molecular weight^5,6^, or perhaps the right surface characteristic descriptors could be found to replace the bulk characteristics in existing chemical models. A multitude of physical and chemical characterizations were cataloged describing a broad range of pristine nanomaterial properties and toxicological endpoints.

As the transdisciplinary work of the nanoEHS field advanced, experiments in complex environmentally relevant systems revealed that nanomaterial characteristics, fate, and therefore impacts, were significantly dependent on the characteristics of their surroundings^7,8^, in some cases as much or more so than the original characteristics of the pristine particle itself. The implications for forecasting both exposure and hazard potential, as well as the performance of engineered nanomaterials, were that this combination of extrinsic and intrinsic characteristics must be known to meaningfully and usefully predict outcomes. The reductionistic approach of chemical modeling would not work for nanomaterials; functional assay approaches to express the effective behavior of the particle in the context of its system were proposed^9^.

The nanoEHS community faced the challenge of how to parameterize and realistically represent the vast number of possible combinations of materials in their various surroundings throughout their lifecycles^10^. This need required a meshing of the epistemologies of natural and physical sciences (describing a complex world) and of engineering (prescribing outcomes and setting system boundaries in order to enable decision-making). Any viable approach must push beyond the impossibility of “it depends” without over-simplifying to the point of irrelevance, while also avoiding complexity paralysis from the sheer impracticality of requiring infinite measurements in stochastic processes. At the same time, the emergence of the nanoinformatics field offered a growing global network of nanoEHS researchers committed to harmonizing experimental design and data curation practices^11–14^.

The novel features and functionalities of nanomaterials that required new approaches toward predicting potential exposure hazards also required new informatics approaches. Publicly available data repositories in bioinformatics and cheminformatics, such as the protein databank (PDB)^15^ and PubChem database^16^, use an informatics structure that focuses on tying static attributes of a chemical to an end result; whether the result be a protein or chemical structure, or functional activity summary. The emerging nanoEHS research indicated that nanomaterial transformations were key to understanding nanomaterial behavior, so the starting characterizations of these materials could not be expected to usefully predict environmentally relevant scenarios. Groups developed new informatics structures to capture the dynamic nature of nanomaterials; resulting in the generation of new repositories shifting from a focus on endpoints, to structures better able to store the occurring changes en route to an endpoint. A leading effort in capturing transformation meta-data was the NanoInformatics Knowledge Commons, or NIKC Database, developed by the Center for Environmental Implications of NanoTechnology (CEINT) to extend upon community efforts to harmonize data such as eNanoMapper^17^. The NIKC belongs to a new generation of repositories developed to structure stored-data to better interrogate the spatial and temporal changes that nanomaterials undergo in complex environmental and biological systems^17–20^.

It was necessary to develop novel data processes to facilitate this new type of repository structure and to enable the curation and processing of data within the NIKC database. Instance Mapping is a guided curation process to adequately capture the experimental and environmental meta-data that are critical to characterizing each step in the transformation of a nanomaterial. The approach of Knowledge Mapping was developed to conceptually capture information needs in a way that could be vetted by both the researcher who generated the data and the curator translating into the NIKC. This framework was further distilled into the Instance Mapping approach; by coupling both data processes, information is translated into systematic, harmonized data structures.

**Fig. 1:**
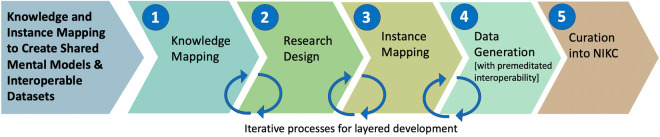
A research workflow demonstrating where in the process Knowledge and Instance Mapping would ideally be used. The circular arrows indicate which steps in the process are frequently revisited and edited over multiple iterations. Knowledge Mapping should be the first step in the process and should be revisited during the design phase. As the research design becomes more refined, the Knowledge Mapp should be updated with any changes or new additions. The Instance Mapping method occurs in the middle of the process as the research design should provide the first Instance Map outline. It is normal for the research plan to change before the end of the experimental process; therefore, any changes made during the experimental process should reflect in the Instance Map, whether that be more or less instances, properties, or media. These mapping methods were originally created for curation into the NIKC database, where the Instance Map is a reflection of the NIKC database structure and can be used as a curation guide for the NIKC Excel spreadsheet before being uploaded into the NIKC database.

Both mapping principles are rooted in centralizing the research question. The Knowledge Mapping method is the more generalized framework, developed as an organizational communication tool for the experimental planning process, for use by all who may ultimately integrate the resulting research (including collaborating researchers, integrators, and data curators), which builds upon systems thinking and systems analysis approaches like the Input-Process-Output (IPO)^21,22^ model. The Instance Mapping method was developed specifically to support data curation efforts into the NIKC structure by visualizing the Instance Organization[al] Structure (IOS) to follow nanomaterial transformations. When used together, Knowledge Mapping and Instance Mapping can enable premeditated integration and identify potential connections and comparisons between results of separate datasets (e.g. Figure 1).

There are other data management frameworks that have been developed or adopted for the needs of nanomaterial and material data. Most notably, ISA-TAB-Nano^23^ which was adapted to fit nanomaterial data needs from the original ISA-TAB format^24,25^. Methods that have been created to try to increase transparency and reusability of nanomaterial and material datasets tend to focus primarily on experimental method development and standardization^26^. The Adverse Outcome Pathways (AOP) framework and subsequently published data analyses demonstrate the essential link between data structure and data usability. The AOP framework links sequential adverse events upon chemical exposure across molecular and system biology levels for environmental risk assessment^27^. While the Instance Mapping method mirrors the importance of linking ‘sequential events’ by tracking material transformations, it can be used across a broader range of fields, and coupled with Knowledge Mapping, link the data with the original research question.

Standard methods have yet to be developed for sharing data across domains^28^ as interdisciplinary collaborations become common, and the need to repurpose data for machine learning and AI grows. Previously developed frameworks are integrated within an informatic infrastructure, usually a data repository^29^. Instance and Knowledge mapping also arose from an informatic infrastructure, but we propose that the concepts are tools individually.

Although these mapping methods were developed to support the NIKC database, Knowledge Mapping and Instance Mapping can be used independently of each other and independently of the NIKC database. The NIKC database was designed to support nanoEHS efforts; however, mapping methods can be used in a variety of cases such as biomedical applications, designing nano-enabled consumer products and for “materialsEHS” including advanced or novel materials. The purpose of this paper is to describe how to create and use Knowledge Maps and Instance Maps through case study examples. This manuscript describes each of these concepts and methods and explains their relationship to supporting the Nanomaterial Informatics Knowledge Commons (NIKC)^30^ and the international collaborative network that enables its growth. Employed together, the approaches shared here support the creation of shared mental models within an experimental team, robust research design, and datasets that are sufficiently contextualized to address nanoEHS research questions and interoperable with the work of others.

## Results

Mapping methods are presented and explained for four experimental scenarios here. Though we also describe how these mapping methods may be used within consortia to coordinate multiple interlinking experiments (in the methods section), in this paper we focus on applying the technique within a single experimental effort, meaning for use within experimental studies being designed and performed by a few people. There are four scenarios encapsulating different types of transformations nanomaterials may undergo based on selected nanodisciplines. With all but one scenario, is a corresponding Knowledge and Instance Map, demonstrating possible ways each map can be constructed based on the scenario. Two scenarios - nanomedicine and consumer products - are hypothetical scenarios inspired by multiple published studies. The other two scenarios are maps made from real published works. These maps were constructed using PowerPoint and Lucidchart; however, any software maybe used to build Knowledge and Instance Maps, including old-fashioned paper and pencil. To conserve space, three scenarios are brief examples of experimental scenarios, only one scenario details a full experimental lifecycle scenario. These decisions were made to demonstrate the broad application of Knowledge and Instance Maps, while also demonstrating the complexity that Knowledge and Instance Maps can convey.

### Application to nanomedicine

**Fig. 2:**
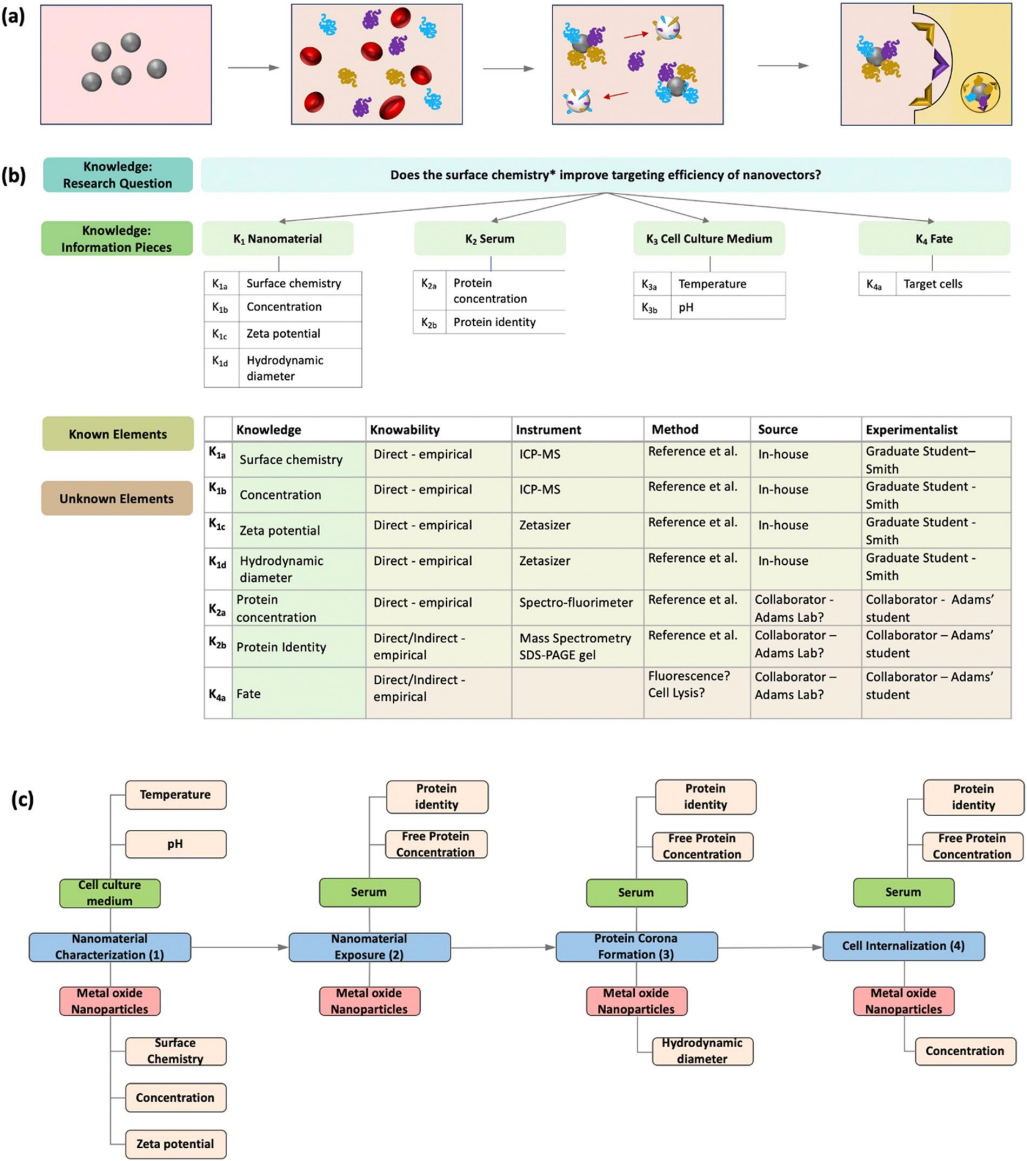
An illustration of (**a**) an example fate scenario of a nanovector used in a medical context. The corresponding **(b)** Knowledge map illustrating the type of knowledge and sub-knowledge pieces necessary to address the stated research question, and the **(c)** Instance map which shows the transformations that occurred during the study. *Note: in this context ‘surface chemistry’ is meant to represent the engineered surface shell, surface functionalization, or surface coating. We understand that ontologically the ‘surface’ has multiple terms with varying definitions.

Innovations in medicine has frequently used advances in nanotechnology for therapeutic and diagnostic purposes. It is well-known that once nanomaterials are intravenously introduced into the body, serum proteins adsorb to the nanomaterial surface forming the protein corona which influences the nanomaterial fate^31–33^. Figure 2a illustrates a simplified *in vitro* scenario of the protein corona formation and resulting nanomaterial fate for a scenario.

### Nanomedicine knowledge map

Knowledge Mapping was created as a method to systematically ensure that experimental designs incorporate the most appropriate conditions and measurements to address the target research question, and in the process, to improve and clarify potential for eventual interoperability with other datasets. Figure 2b shows the planning process for an example corona-nanomaterial fate experiment. The Knowledge Map consists of three sections: 1) the research question, 2) the knowledge pieces needed to answer the research question, 3) and a table of the known and unknown experimental planning elements. In this scenario, the researcher is investigating whether the nanomaterial’s surface chemistry (e.g. surface coating) increases uptake of the nanovectors into the target cell.

To address this research question, the researcher needs characterizing knowledge on the nanomaterial and the surrounding environment (e.g. the media – serum and cell culture medium), which may influence corona formation. Finally, the researcher needs to know if cell uptake is occurring, and if so, in which cells can the nanovectors be found. Therefore, this Knowledge Map has four main knowledge pieces linked with supporting informational characterizations needed to answer the research question. Each knowledge piece is given an identifier, for example, the nanomaterial is given the identifier K_1_. The supporting characterizations are linked to each knowledge piece in a table, which also assigns each sub-knowledge piece an identifier. For example, the researcher has identified the zeta potential to be an important piece of information to know to address the research question. The zeta potential has been given the identifier K_1c_, indicating that it is a sub-knowledge piece of the nanomaterial.

The final component of the Knowledge Map is a corresponding table detailing the experimental planning process. In practice, the table should exist in a format easily accessible to members of the project, such as on a cloud service. The table may be composed of any variables pertaining to any needs necessary to address the research question. In the corona scenario, the variables chosen relate to planning how each sub-knowledge piece will be measured. The table also indicates which elements are known and which elements are unknown still needing a final decision.

### Nanomedicine instance map

**Fig. 3:**
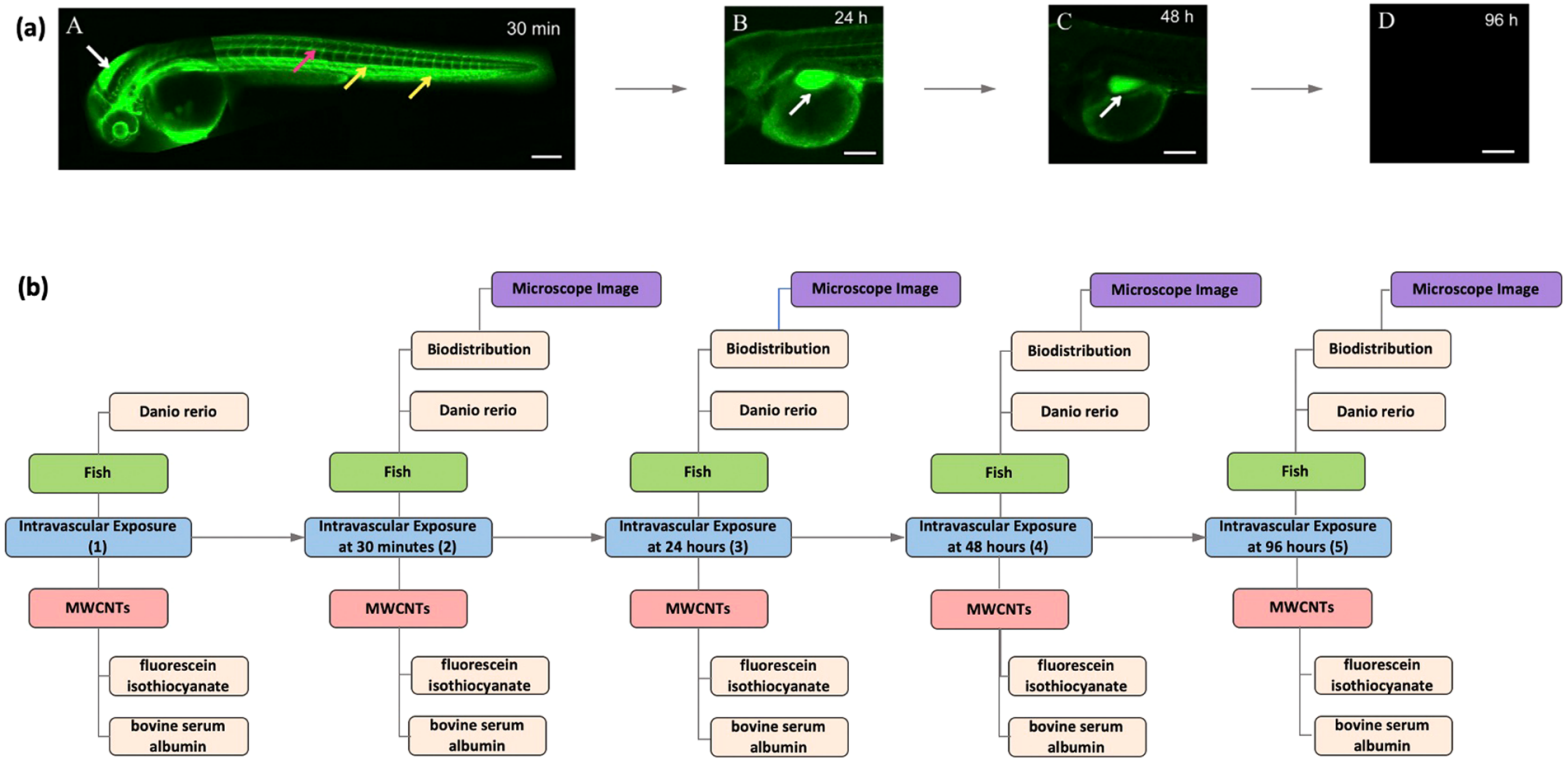
Microscope images* **(a)** showing changes in biodistribution of surface-modified MWCNTs during depuration of the nanomaterial with the corresponding **(b)** Instance map. *The microscope images were not produced by the authors of this paper and were originally published by Cheng *et al*. and republished here with permission.

Figure 2c shows an Instance Map drawn for the fate experiment illustrated in Fig. 3a. This Instance Map has four instances as the nanomaterial undergoes three nanomaterial transformations. In the first instance, the nanomaterials are characterized in cell culture media. There are measurements characterizing both the cell culture medium, temperature and pH, and the material, surface chemistry, concentration, and zeta potential, which are categorized as properties of the material and medium. These characterized nanomaterials undergo a transformational change when they are added to serum because the nanomaterial’s surrounding environment has changed, resulting in the second instance.

In the second instance, the new medium has two measurements associated with it: identification of the proteins in the serum and the protein concentration. In the third instance, the serum proteins have adsorbed onto the nanomaterials’ surface, forming the corona. A transformation has occurred because properties of the combined system have changed – in this case, properties of both the medium and the material. With the formation of the corona, we would expect the concentration of serum proteins to decrease, along with a potential change in the identity of the serum protein. Depending on the characteristics of the corona, we would also expect the hydrodynamic diameter of the nanomaterial to change. In the fourth and final stage of this Instance Map, the nanomaterial maybe internalized within a cell based on the identity of the corona and receptors on the membrane surface, which could be determined based on the detection of a change in the nanomaterial concentration remaining in the serum.

### Application to nanotoxicology

As previously stated, these mapping methods were originally designed to accompany data processes for curation into the NIKC database architecture, which was designed with the intent to enable consistent, comparable data capture for nanomaterials in complex systems^30^. The example scenario in Fig. 3a is from a published manuscript^34^ curated into the NIKC, and therefore, uses real data published by non-CEINT authors. Figure 3a visually follows an *in vivo* depuration of fluorescently labeled multi-walled carbon nanotubes (MWCNTs) from zebrafish (*Danio rerio*) larvae. The microscope images featured in Fig. 3a were originally published by Cheng *et al*.^34^ and republished here with the authors’ permission, while the size of the original images have been changed, the content of the images have not been altered. The entire experimental process published in the original manuscript is not represented here. Since this example scenario uses real data not published by the authors of this paper, there is no accompanying Knowledge Map for this scenario.

### Nanotoxicology instance map

Figure 3b is an Instance Map detailing nanomaterial distribution changes during a vertebrate depuration phenomenon occurring over multiple time points^34^. This Instance Map represents five instances and four transformations. The first instance represents the initial exposure. The material in this map is the MWCNTs used in this depuration experiment, which have two identified surface chemistries listed as properties of the nanomaterial. The “medium” throughout the Instance Map is the exposed zebrafish and does not change throughout the experimental process. The transformations represented here are the internal MWCNTs distribution changes, which corresponds with timepoint measurements starting with the initial nanomaterial exposure and incremented up to 96 hours post exposure. For every timepoint after the initial exposure, a new measurement is taken representing the internal distribution of the nanomaterial, which is listed as a property of the medium. The microscope image itself is categorized as the supplementary characterizing the distribution property.

### Application to nanocomposite materials in consumer products

**Fig. 4:**
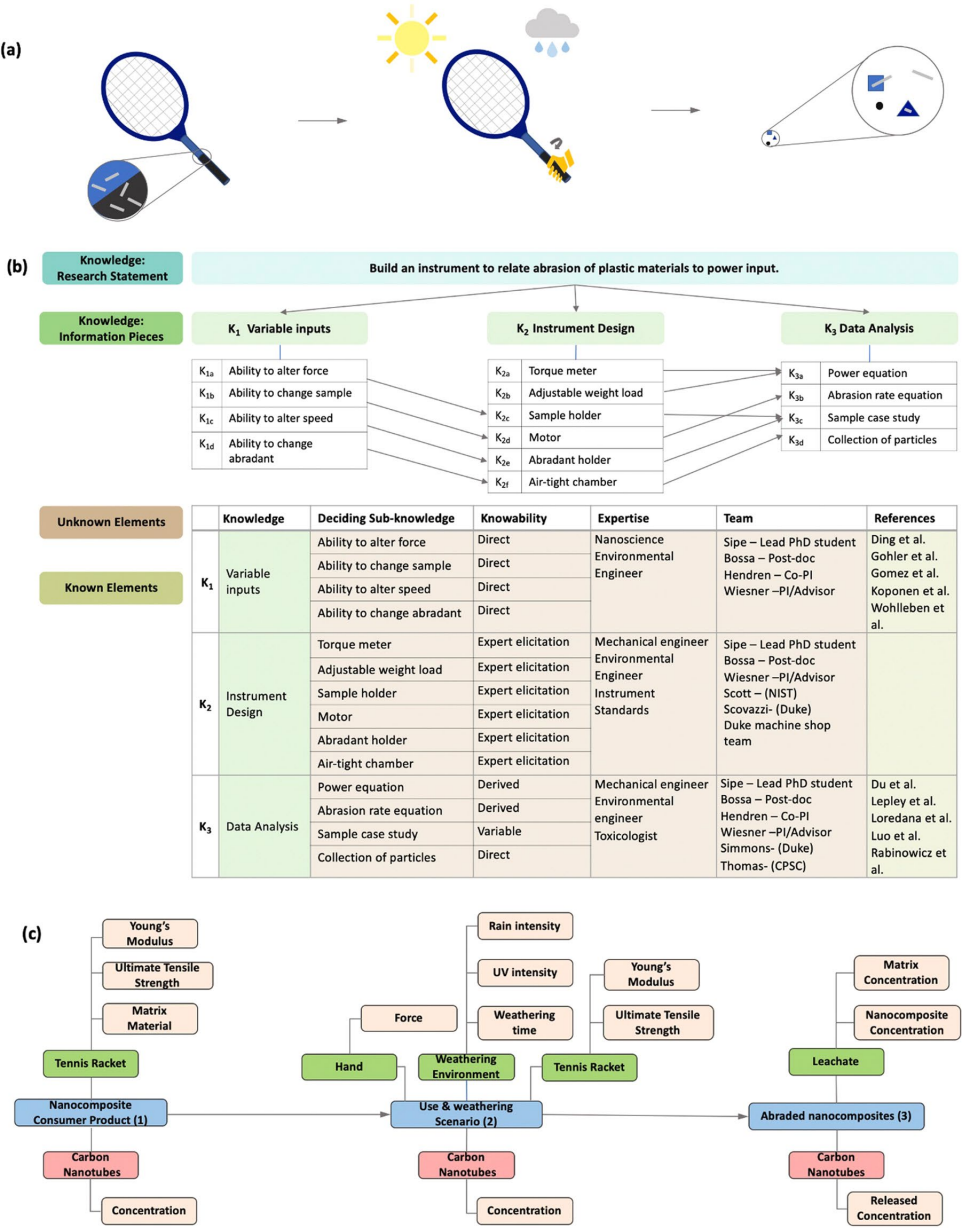
An illustration of **(a)** nanocomposite exposure through consumer product use scenario inspired by work performed at CEINT in collaboration with the CPSC. The resulting Knowledge Map **(b)** shows the planning involved in building the abrasion instrument built by the Wiesner lab at Duke with consultation from NIST, CPSC, and USACE. To the authors’ knowledge, it is the first abrasion instrument built to correlate power input during the abrasion process making it possible to model realistic mechanical product breakdown scenarios. The resulting Instance Map **(c)** of the product use scenario **(a)** was made possible by the construction of the abrasion instrument **(b)**.

Many people are exposed to nanomaterials through the consumer products that are purchased on a daily basis^35,36^. Nanomaterials are typically added to consumer products to enhance product performance or durability. The example in Fig. 4a is a simplified example of nanomaterials added to sports equipment, and nanomaterials generated and released in a product usage scenario. This hypothetical scenario is based on experimental data generated through research within the CEINT network^37,38^.

### Nanocomposite materials knowledge map

The Knowledge Map for this scenario does not detail the planning of the hypothetical consumer product scenario illustrated in Fig. 4a. Instead, a Knowledge Map was created to aid the design and construction of the abrasion instrument built^39^ at Duke University that would be used to model product use of a consumer product. Designed and constructed in collaboration from the National Institute for Standards and Technology (NIST), the Consumer Product Safety Commission (CPSC), and the U.S. Army Engineer Research and Development Center (ERDC), the Duke abrader was the first instrument built capable of correlating power input to abrasion of plastic and nanocomposite materials, which is essential for modeling abrasion rates of consumer products, such as the example presented in Fig. 4a.

The first step in constructing the Knowledge Map is clear articulation of the research question or objective, which in this case was to how to build a machine capable of relating power input to the abrasion process. There were three main knowledge pieces to consider when building the instrument: 1) the variable inputs, 2) the design of the instrument, 3) and how to analyze the data generated from the instrument. The sub-knowledge pieces are linked in tables below each knowledge piece, similar to the previous Knowledge Map, each knowledge and sub-knowledge piece is given an identifier. Unlike the previous Knowledge Map, there are arrows present to illustrate the interlinking purpose of each sub-knowledge piece toward the overall construction of the instrument.

The Knowledge Mapping process enabled effective design of the instrument such that research objectives could be met, while also enabling clear thinking and communication across the collaborators of this design and construction endeavor (Duke, CPSC, NIST, and US Army ERDC). The Knowledge Mapping table of knowns and unknowns describes which parts of the planning process were known a priori, and which parts were decided upon during design and construction phases. The table also shows the five different fields of expertise necessary to build the instrument. The people listed in the table were directly involved in the construction and determining the data analysis process.

### Nanocomposite materials instance map

Figure 4c illustrates an Instance Map created for the sports equipment product-use scenario consisting of three instances and two transformations. In the first instance, a tennis racket is manufactured with carbon nanotubes embedded in the product matrix. The measurements listed as properties of the tennis racket, which is the medium for the carbon nanotubes in this scenario, characterize the product’s durability. Use of the product has resulted in a transformation and the second instance.

The second instance is more complex than previous examples because it has multiple media characterizing this instance. In the use scenario, a consumer is using the tennis racket in various environmental conditions. The player is applying abrasive force to the tennis racket while playing tennis. At the same time, the racket is being weathered due to UV and rain exposure. It is possible that the wear on the tennis racket may have made the product more brittle changing some of the product material’s durability properties. In the final instance, the abraded product is collected as a leachate to identify the material forms (e.g. free matrix, free nanomaterial, composite material) that is abraded from the racket.

### Application to ecotoxicology

**Fig. 5:**
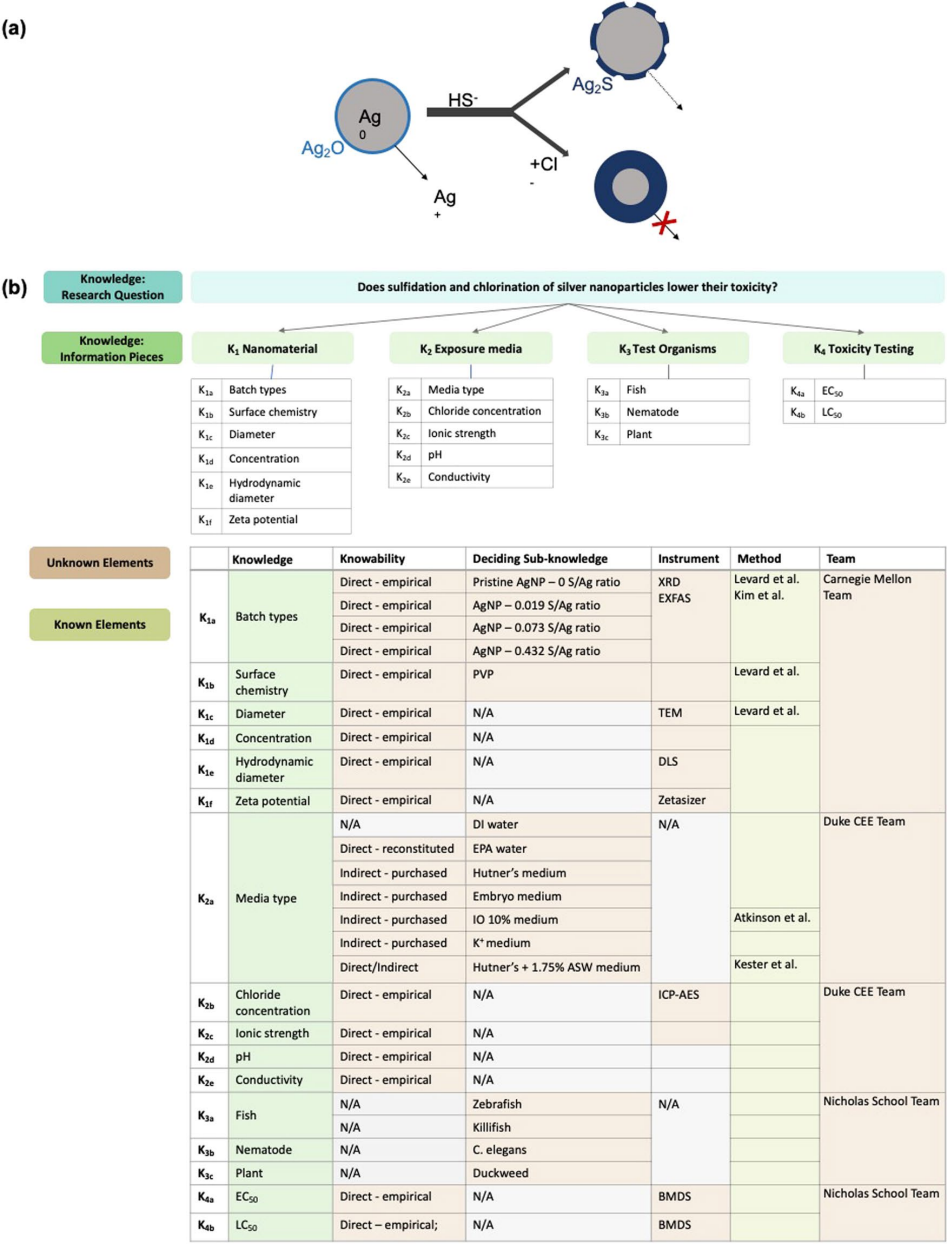
An illustration **(a)** of an ecotoxicity study, conducted by CEINT members, of organisms in reconstituted environmentally relevant conditions exposed to silver nanomaterials. The image is based on the original graphical abstract in Levard *et al*. The corresponding Knowledge Map **(b)** of the decisions involved in planning the experimental study.

**Fig. 6:**
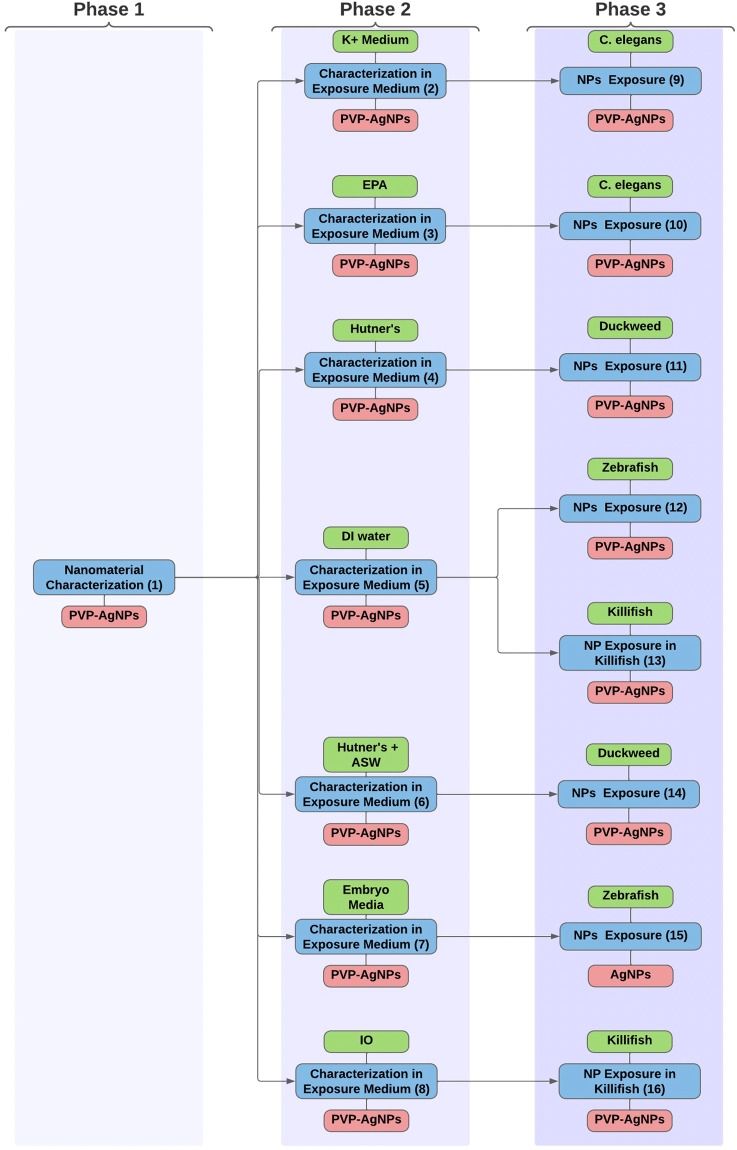
The resulting Instance Map of the ecotoxicity study testing the effect of sulfidation and chlorination of silver nanoparticles on detrimental toxicity to organisms. The full Instance Map including properties can be found in the supplementary (e.g. Supplementary Information 1).

The final Knowledge and Instance Maps are the most comprehensive created in this publication, encompassing an intricate experimental study by Levard *et al*. (2013). This experimental study looks at the effect of sulfidation and media composition on attenuating silver toxicity in exposed organisms (e.g. Figure 5a; remade with permission)^40^. Four batches of silver nanoparticles characterized in seven different aqueous media are exposed to four organisms. Each batch of silver nanoparticles has a different level of sulfidation. An Instance Map should only track the transformations of one nanomaterial; therefore, there should be four Instance Maps made, one for each batch of silver nanoparticles. For simplicity, only one Instance Map of one silver nanoparticle batch is shown in Fig. 6, because each silver nanoparticle batch goes through the same set of experiments.

### Ecotoxicology knowledge map

The experimental process for this set of studies requires an incredibly complex Knowledge Mapping exercise, due to the number of batches of nanomaterials (four), aqueous media conditions (seven), and different organisms tested (four). In the context of this complexity, it is noteworthy to share that the Levard *et al*. publication^40^ was chosen in part because two of the authors on that publication are also authors on this current publication, and could therefore provide insight into the planning process. This can be critical for complete and accurate Instance Map construction, because the role Knowledge (and Instance) Maps play is to visually present datasets inclusive of all meta-data that may be relevant to nanomaterial transformations and properties. Often, not all of these meta-data are included in publications, or are inconsistently shared within or across publications. Therefore, communication with the researchers is frequently needed to enable a complete map, highlighting the value of adopting Knowledge and Instance Mapping as a consistent practice across studies of nanomaterials in complex environments.

The researchers were testing whether sulfidation levels of silver nanoparticles and the chloride concentration of the aqueous medium could attenuate the toxicity seen from pristine silver nanoparticles – this is captured as the target research question to start the Knowledge Map (e.g. Figure 5b). Sulfidation and chlorination processes were chosen based on relevance to transformations silver nanoparticles would undergo in the environment.

Four main knowledge pieces were necessary to address this research question: 1) the nanomaterial, 2) exposure media, 3) test organisms, and 4) toxicity. The corresponding sub-knowledge pieces are listed in tables below with unique identifiers. The table lists the known and unknown elements of the experimental planning process. In this case we are retro-fitting a Knowledge Map that was not actually created as part of the experimental design process; however, for the purposes of this paper we note how the KM activity could support the design process. For example, how many batches of silver nanoparticles should be used? What levels of sulfidation should be chosen? Should multiple organisms be tested to provide evidence that toxicity mitigation is not just a trend for one species system? What type of organisms should be tested? An experimental study of this size also requires multiple collaborating partners. This project was performed between two universities and five labs.

### Ecotoxicology instance map

Figure 6 shows an Instance Map consisting of 16 instances and 15 transformations. This is an abbreviated Instance Map containing only the instances, media, and material. The full Instance Map with all characterizing properties can be found in the supplementary information of this manuscript (e.g. S1). The map in Fig. 6 is divided into three sections for clarity because of the number of instances; each section represents a different phase of the experimental study.

The first phase of the study contains one instance which represents the initial characterization of silver nanoparticles after being synthesized in-house. The second phase of the study contains seven instances in which the silver nanoparticles are characterized in different aqueous exposure media. In the experiment, the synthesized silver nanoparticles are characterized in seven exposure media (DI water, EPA water, Embryo medium, Hutner’s medium, Hutner’s + 1.75% ASW, K^+^ medium, 10% IO medium) with different ion compositions. There are seven instances because each unique nanomaterial-aqueous media combination has its own set of media characteristics, e.g. ionic strength, and nanomaterial characteristics, e.g. hydrodynamic diameter. Seven transformations have occurred because the medium, or environment of the nanomaterial, has changed with each dilution into a different aqueous media.

The third phase of the experimental study is the nanomaterial exposure to four model organisms to evaluate toxicity responses to silver nanoparticles in each of the seven differing media compositions. There are eight instances in this phase representing the unique organism-aqueous medium pairings: zebrafish-DI water, zebrafish-embryo media, killifish-DI water, killifish- IO 10% medium, *C. elegans*-EPA water, *C. elegans*-K^+^ medium, Duckweed-Hutner’s medium, and Duckweed-Hutner’s + 1.75% ASW medium. Each of these organism-aqueous medium pairings follow the previous instances when the silver nanoparticles were first characterized in the aqueous medium.

## Discussion

We present two data processes - Knowledge and Instance Mapping - that adequately capture the metadata necessary to create more cohesive and interoperable datasets within nanoscience fields. We are proposing that the Instance Mapping method allows for someone to 1) get an overview of the entire experimental design in one diagram, 2) organizes the data such that characteristics of the nanomaterial are linked to specific moments or environments, 3) and that having an Instance Map is an important tool for collaborative projects and decision making processes such as policy and regulatory decisions. We are also proposing that using the Knowledge Mapping method can 4) clearly connect the essential information pieces to the research question which 5) for multi-phased and multi-disciplined collaborative projects clarifies roles, responsibilities, and project timelines. Secondary analysis of data involving machine learning algorithms may require collating a large dataset from multiple researchers produced during evolving phases of advancing technological and knowledge timelines. We are proposing that data scientists can use 6) Knowledge and Instance Maps describing experimental data to determine whether the data should be curated into the larger dataset based on the secondary analysis research question.

This determination can be based upon a quick look at the metadata in the Instance Map and at which stages in the process the metadata was recorded. For example, we have previously published our criteria for curating data into the NIKC database based upon our data quality and data completeness needs, which will vary from project to project based on the research question. We previously described that “[m]any published studies of nanomaterials inadequately characterize either the nanomaterial or the medium, or in some cases, both. To ensure high-quality data from literature sources, we curate papers with nanomaterial characterizations performed by the researchers themselves or their partners…. We also look for papers that characterize the exposed medium of the nanomaterial, whether that be a cell culture medium or an organism.” It was also important for our needs that the nanomaterial be characterized in the exposure aqueous medium. Instance Maps were necessary not just to help organize data for NIKC curation, but also to clearly see what metadata was recorded, when measurements were taken, and by whom, which then determined whether we incorporated the data into the NIKC.

As nano-disciplines integrate machine learning and other advanced statistical analysis toward understanding and predicting nanomaterial behavior, it is imperative to design research questions and associated experiments such that the data they generate are premeditated to be interoperable and less disparate. Standardized visualizations can ensure that researchers are working with a shared mental model of research and resulting data, and that they can quickly convey the research question and experimental design to data analysts^41^. These practices can help determine whether datasets should or can be collated, and can also help analysts to connect seemingly unrelated datasets due to ontology choices.

Data generated from experimental results ideally live multiple lifespans beyond their original use and serve broader intentions than the original experimental research question that motivated their collection^42^. Some research questions require the discernment of trends and patterns from experimental or observational data generated internationally to create ‘big’ datasets (e.g. as evidenced in climate change research, which is built upon vast datasets collected globally over decades^43^). The practice of assembling vast datasets generated by internationally diverse researchers is standard in scientific disciplines. The nanoEHS community is no exception with the added complexities of the dynamic nature of nanomaterials in complex environments, and the inherent requirement of convergence across multiple scientific disciplines.

Creating shared data processes that can be applied independently of a database is essential for enabling consortia science which may span multiple universities across multiple countries with conflicting data sharing laws. Knowledge Mapping is currently being used in active collaboration projects to communicate proposed research questions and logistics on performing experiments at multiple institutions. This author team has applied Instance Mapping broadly across internal collaborations within CEINT, with the French CEREGE (European Center for Research and Teaching in Environmental Geosciences), and with broader CEINT network collaborators, such as the EU-based NanoFASE and NanoCommons projects. In practice, we found Instance Maps helped to complete the data curation and translation processes by providing a more complete representation of experiments. We presented these mapping methods as they were originally created and are still used in-practice; however, NanoCommons has taken the Instance Mapping baton and transformed it through the development of an online Instance Mapping Tool (https://instance-maps.stage.sevenpastnine.com). Please contact Thomas Exner for access). The use of Knowledge and Instance Mapping will be discussed in future publications with our collaborators’ perspectives.

The nanoEHS community is actively working to make data more interoperable through the adoption of the FAIR principles. Work produced out of communities of practice, such as the National Cancer Institute’s Nanotechnology Working Group’s (NanoWG) further development of the ISA-TAB-Nano^23^ spreadsheets and publications, provide guidance on how to FAIRly^44^ implement informatics in nanoEHS. Knowledge and Instance Mapping may also be useful tools toward understanding other emerging contaminants, such as microplastics. Microplastics frequently break down into nanoplastics through weathering and abrasion processes which may facilitate their transport in the environment and increase their toxicity through chemical leaching^45–47^. More broadly, these tools are being adopted and applied to enable interlinked convergent research to address wicked problems such as global sustainability of phosphorus cycling.

Science is by nature a collaborative enterprise requiring data openness and sharing practices to advance and develop, which have traditionally existed in the form of peer-reviewed publications. Nanomaterials present unique challenges to protocol standardization and regulation due to their dynamic nature. Capturing nanomaterial transformations in exposed systems, standardizing testing protocols, and explicitly recording nanomaterial synthesis^48^ methods are essential for understanding how to create safe nanomaterials. Safe nanomaterials are a growing imperative as the evolution of applied nanotechnology is continually expanding into various aspects of society through avenues such as agriculture and consumer products. Knowledge and Instance Mapping are data processing methods that can co-evolve with nanotechnology as it continues to transition. Knowledge and Instance Mapping are purposely lacking in rigidity to accommodate differing transition rates across various nanofields. An instanced-based approach to organizing data is well suited to the needs of evolving needs of nanoEHS from engineered and advanced materials, as well as to the increasing challenges of natural and incidental materials released with the advent of climate change. Although, we have presented these methods independently of the CEINT NIKC (pronounced ‘saint nick’) database, we encourage all who would like to use these mapping methods tools in alignment with the NIKC to contact Mark Wiesner for access.

## Methods

### Knowledge mapping

**Fig. 7:**
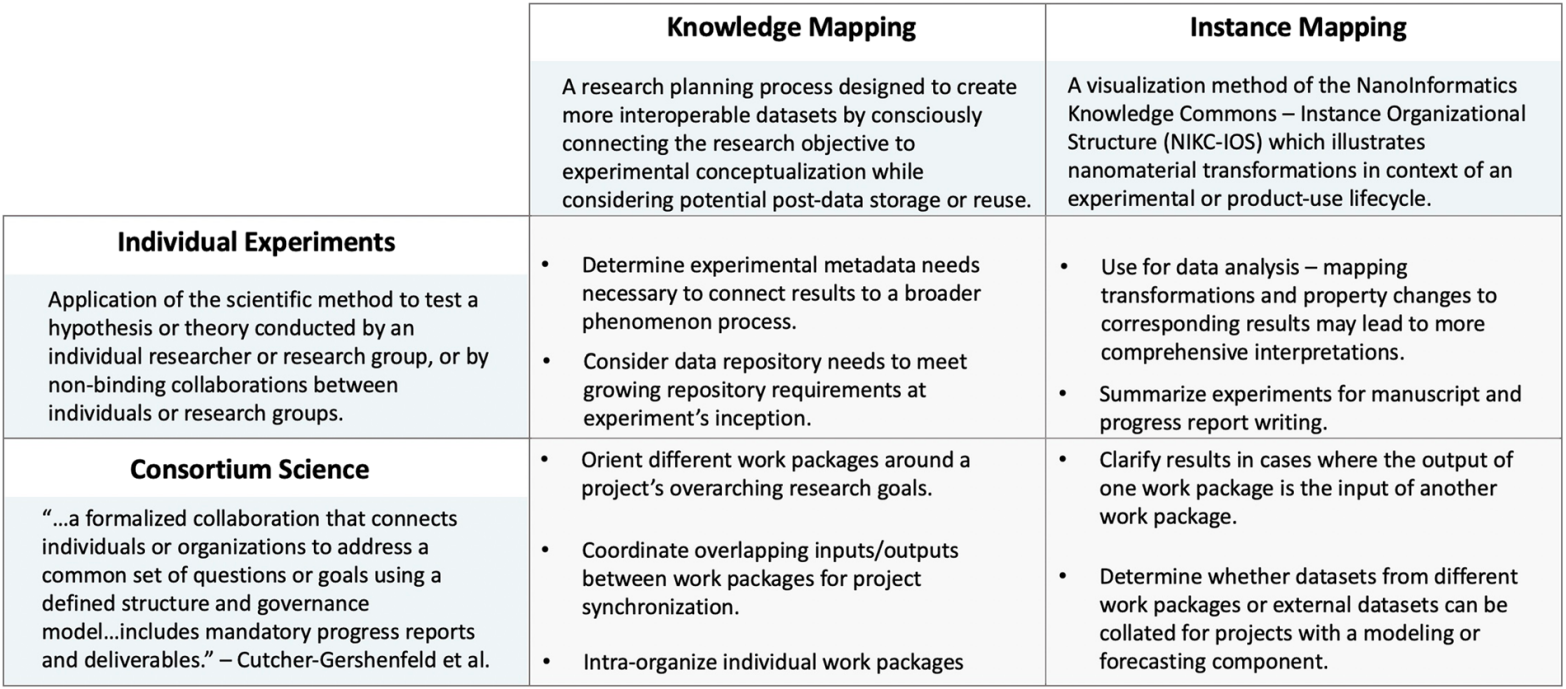
We describe how Knowledge and Instance Mapping can be applied on both a micro and macro level. In this paper, we focus primarily on micro – individual – applications of these mapping methods but wanted to clarify mapping method applications within consortia^49^. The outer blue boxes define the terms used within context of this publication. The center gray boxes list some of the applications of Knowledge and Instance Mapping within consortia and individual settings.

Knowledge Maps capture the conceptual schema of all the pieces of information that a research process would ideally generate, as well as all of the pieces of information that would be required to feed into the research. The explicit articulation of this “wish list” of knowledge – intentionally detached from the limitations of existing data – enables researchers to holistically consider the collection of information or knowledge needed to address a research question or questions. Knowledge Mapping can be applied on a micro (e.g. intra- or inter-laboratory experiments) and macro (e.g. inter-institutional coordination within a consortium) level (e.g. Figure 7). On a macro level, Knowledge Mapping can enable connection across related efforts that might share knowledge inputs or intended outputs or that might be interdependent on one another, and therefore enable researchers to premeditate the harmonization of knowledge across all efforts. There are four components of Knowledge Mapping: identifying output knowledge, identifying input knowledge, categorizing the information pieces in terms of “knowability”, and sourcing the knowledge.

The components of the output knowledge need to be determined before identifying the components of input knowledge. Output information includes new knowledge the research effort (experiment, model, qualitative analysis) is intended to generate. It can be helpful to start with the research question, then articulate what new knowledge is expected to be gained through the course of the research process. New knowledge can include endpoint measurements of functional assays, or a new model (and its parameters) constructed to explain process phenomena. To ensure that one includes all the pertinent output knowledge, it may be useful to produce a map to structure thinking on anticipated data reuse or combination and the implicated parties. Within a consortium, data generated from experimental results may be collated with other datasets to answer a broader modeling question. Data produced from individual experiments may need to be stored in a repository before being published, which may have specific metadata requirements.

The input knowledge pieces can be determined by traveling “upstream” from the desired output knowledge to identify the pieces of information needed to acquire the new knowledge that is being sought. A familiar reframing for informaticians is to identify and categorize the data and metadata needed. For example, when performing the attachment efficiency (α) functional assay, the desired information is an (α) value; however, properties of the nanomaterial and media are needed to understand the contributing factors to the resulting (α) value. When considering data reuse, metadata needed by the experimentalist to interpret their results may be different from the metadata needs of a modeler. The experimentalist may need to collect more measurements to meet the metadata needs of a modeler.

Once the information, which we refer to in discreet form as “knowledge pieces” or “sub-knowledge pieces”, have been identified, the next step is to assign each one into a category that best represents its “knowability” and general type. The language used here is intentionally generalized to be inclusive of many data types and methods. This is the point at which the broad concept of all the idealized information one would need is then evaluated and fit into the constraints of our information reality. Some will be “knowable” through empirical data. Some might be “knowable” theoretically, as estimated by a model, or probabilistically projected. Some might be represented by proxy with another knowable piece of information, or projected by expert elicitation. Others might be unknowable for the purposes of the research effort for a variety of reasons including confidential business information, technical limitations, or budgetary constraints.

The final step in Knowledge Mapping is to be specific in the sourcing of all the knowable information. For each piece, this step prompts the researcher to drill down to the literature source, the specific assay and method being used for experimental data, the models and theories used to estimate, the particular people whose expert opinion is being elicited, the proxy variable being plugged in because the one we wish was available is currently unknowable/immeasurable, etc. It is at this point that experimental and potentially integration decisions can be made in a systematic way by moving through the list.

If multiple methods or assays are available to get one knowledge piece, which methods are most appropriate for a given research question? Specifying what spatial and temporal scales are necessary for the experimental project will also help drill to a detail level that is actionable and broadly legible across interested collaborators at this point. For example, weathering experiments may need six months of environmental exposure to adequately replicate real-world examples; whereas, an exposure-toxicity study may only require a week experiment time depending on whether exposure is acute or chronic. This step in particular should be iteratively revisited, and ideally used as a communication tool within teams of mentor/mentees and collaborators generally.

### Instance mapping

**Fig. 8:**
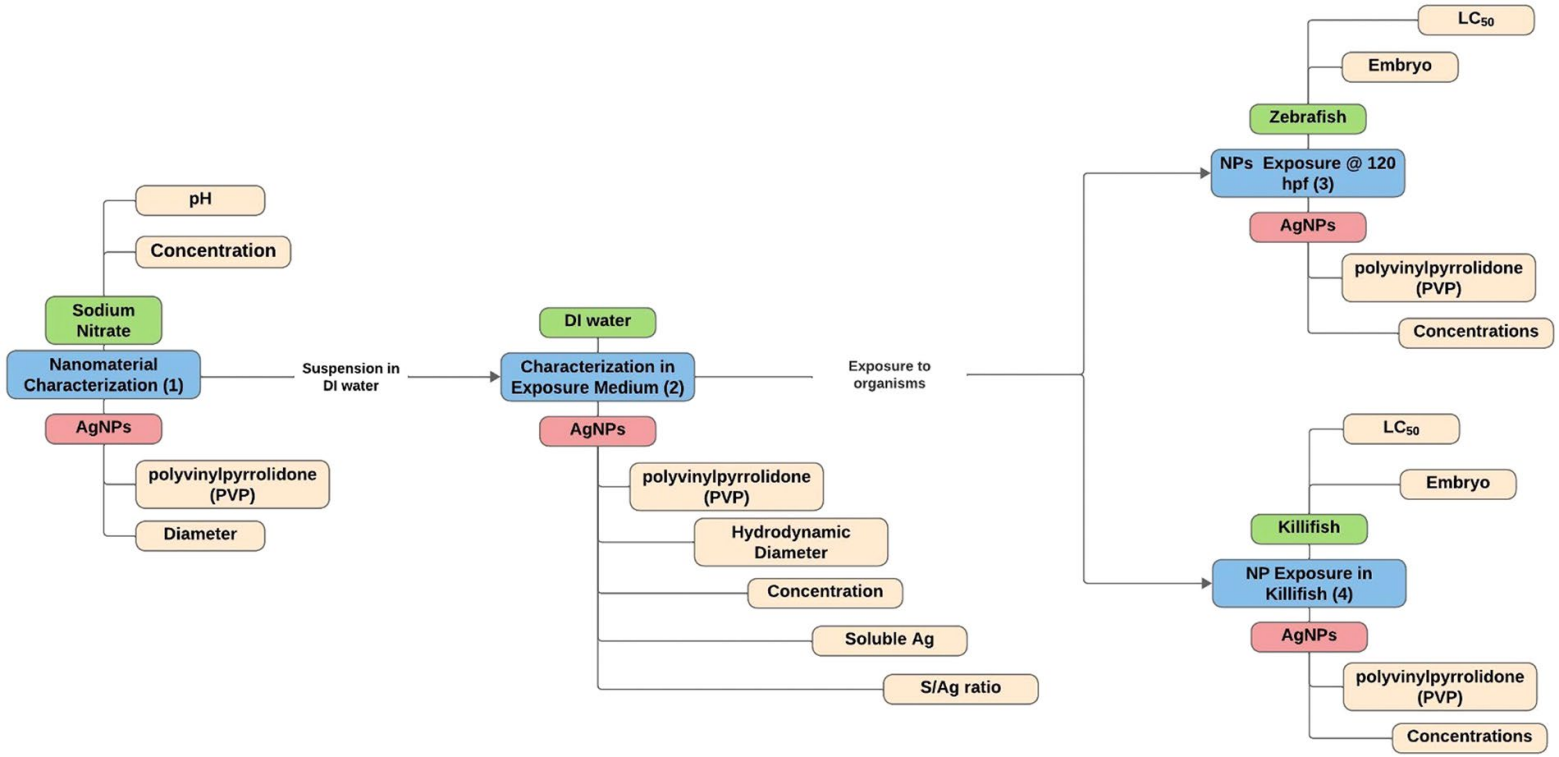
Understanding how to read Instance Maps is about learning how to follow the flow of material transformations through an experimental lifecycle represented here from left to right chronologically. Here we show a snippet of the full map, which can be found in the SI. By following the flow of arrows connecting each instance, we can follow the general flow of transformations. The arrows themselves represent a transformation. There are two transformations here: (1) the suspension of silver nanoparticles in the exposure medium, (2) and the exposure of the organisms to the nanoparticles.

An “instance” is defined by a point of time and/or a “place” (e.g., a beaker or a tissue). The “place” is defined in part by the properties of the medium making up that place (e.g., ocean water, blood plasma). In any given instance, the properties of a material of interest (e.g., carbon nanotube) are defined and recognized to potentially vary with the properties of the medium and over time. The defining characteristics at a moment in time may change over the course of multiple nanomaterial transformations. The medium was purposefully given a broad definition to accommodate the many formulations and uses of nanomaterials across fields such as the biological and environmental sciences. An instance may be described by multiple media; for example in cell line exposures, the media may include the cell culture media and the cells themselves. Or for plant uptake experiments media may include nanomaterial concentration distribution between the soil, roots, or leaves of the plant. However, there should be one material per Instance Map. An example on how to read an Instance Map can be found in the figure below (Fig. 8).

Instance Maps were developed as a visual manifestation of the structural schema of the NIKC-IOS within the CEINT NIKC database, articulating the snapshot of the nanomaterial properties alongside the co-occurring surrounding medium properties. Instance Maps were developed to visually track nanomaterial transformations in diverse environmental and biological systems, to express the value of these metadata, and to enable curation. The NIKC database was designed to store raw and published experimental data. When creating an Instance Map for raw data, protocols associated with the experimental design should be reviewed to identify any points at which the media surrounding the material would have changed and new measurements were taken, to trace and contextualize any transformations that have occurred. When creating an Instance Map from published literature that is not one’s own work, then the abstract, materials and methods, and the results or discussion should be referenced. Either origin point follows the same basic steps.

The process begins by identifying each nanomaterial transformation that occurs during the experimental lifecycle, these will represent the instances of the Instance Map. To determine if a new instance has occurred, one must identify if a change has occurred that has transformed the properties of nanomaterial or the medium (media). For example, a transformation of properties defining the medium may include incorporating nanomaterials into clothing for its antibacterial properties. The number and type of microorganisms found on the clothing after wear may change with addition of the antibacterial properties lent to the fabric by the nanomaterials. Additional examples of instance changes include transformations of pristine nanomaterials to surface-modified nanomaterials, which may be caused by engineered or natural processes. Natural processes may include sulfidation in environmental surroundings, or the fluctuating identify of the protein corona in biological surroundings.

Properties capture the measurement and endpoint data gathered during the experimental lifecycle. Properties should be linked to either the medium or material in the Instance Map. Examples of properties linked to the material may include the diameter, shape, or surface area of the nanomaterial when its physical state is as powder. If the nanomaterial undergoes a transformation that changes its physical state to a suspension, the properties of the nanomaterial may have also changed to include hydrodynamic diameter, zeta potential, and concentration. Examples of properties linked to the medium for whole organism toxicity studies may include the organism’s Latin name, the sex, or lifestage. The supplementary is used as a placeholder for visual data including microscope images, which can be considered as additional property descriptors. If using Instance Maps in combination with Knowledge Mapping, the sub-knowledge and knowledge pieces may become Instance Mapping categories, depending on the nature of the Knowledge Map (e.g. conceptual planning of an experimental design versus a prediction model) and the “knowability” of the knowledge and sub-knowledge pieces.

### Supplementary information

Supplementary Information

## Data Availability

Data sharing not applicable to this article as no datasets were generated or analyzed during the study.

## Code availability

No custom code was used to develop the data processing methods described in this article.
