# Supplementary material for: Knowledge and Instance Mapping: architecture for premeditated interoperability of disparate data for materials
Source: Sci Data. 2024 Feb 6;11:173. doi: 10.1038/s41597-024-03006-8 (PMC10847415; doi:10.1038/s41597-024-03006-8)
Supplement: Supplementary file 1 — Supplementary Information [file 41597_2024_3006_MOESM1_ESM.pdf]

Supplementary Information

List of Figures

Figure S1 Full Instance Map of ecotoxicology study…….…………2
Description of full ecotoxicology study…….…..………….3

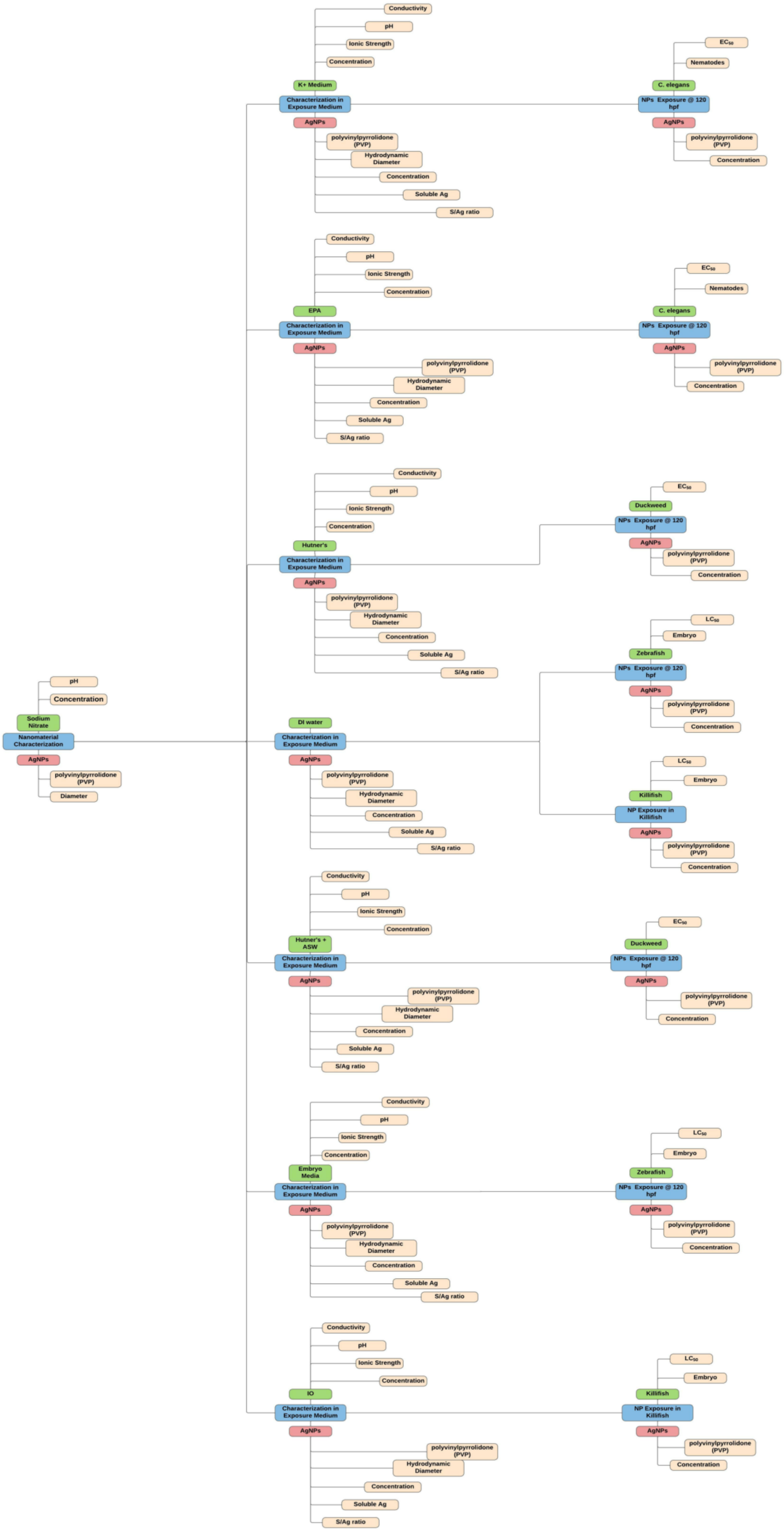
Sodium Nitrate
pH
Concentration
Nanomaterial Characterization
AgNPs
polyvinylpyrrolidone (PVP)
Diameter
K+ Medium
Conductivity
pH
Ionic Strength
Concentration
Characterization in Exposure Medium
AgNPs
polyvinylpyrrolidone (PVP)
Hydrodynamic Diameter
Concentration
Soluble Ag
S/Ag ratio
C. elegans
EC_{50}
Nematodes
NPs Exposure @ 120 hpf
AgNPs
polyvinylpyrrolidone (PVP)
Concentration
EPA
Conductivity
pH
Ionic Strength
Concentration
Characterization in Exposure Medium
AgNPs
polyvinylpyrrolidone (PVP)
Hydrodynamic Diameter
Concentration
Soluble Ag
S/Ag ratio
C. elegans
EC_{50}
Nematodes
NPs Exposure @ 120 hpf
AgNPs
polyvinylpyrrolidone (PVP)
Concentration
Hutner's
Conductivity
pH
Ionic Strength
Concentration
Characterization in Exposure Medium
AgNPs
polyvinylpyrrolidone (PVP)
Hydrodynamic Diameter
Concentration
Soluble Ag
S/Ag ratio
Duckweed
EC_{50}
NPs Exposure @ 120 hpf
AgNPs
polyvinylpyrrolidone (PVP)
Concentration
DI water
Characterization in Exposure Medium
AgNPs
polyvinylpyrrolidone (PVP)
Hydrodynamic Diameter
Concentration
Soluble Ag
S/Ag ratio
Zebrafish
LC_{50}
Embryo
NPs Exposure @ 120 hpf
AgNPs
polyvinylpyrrolidone (PVP)
Concentration
Killifish
LC_{50}
Embryo
NP Exposure in Killifish
AgNPs
polyvinylpyrrolidone (PVP)
Concentration
Hutner's + ASW
Conductivity
pH
Ionic Strength
Concentration
Characterization in Exposure Medium
AgNPs
polyvinylpyrrolidone (PVP)
Hydrodynamic Diameter
Concentration
Soluble Ag
S/Ag ratio
Duckweed
EC_{50}
NPs Exposure @ 120 hpf
AgNPs
polyvinylpyrrolidone (PVP)
Concentration
Embryo Media
Conductivity
pH
Ionic Strength
Concentration
Characterization in Exposure Medium
AgNPs
polyvinylpyrrolidone (PVP)
Hydrodynamic Diameter
Concentration
Soluble Ag
S/Ag ratio
Zebrafish
LC_{50}
Embryo
NPs Exposure @ 120 hpf
AgNPs
polyvinylpyrrolidone (PVP)
Concentration
IO
Conductivity
pH
Ionic Strength
Concentration
Characterization in Exposure Medium
AgNPs
polyvinylpyrrolidone (PVP)
Hydrodynamic Diameter
Concentration
Soluble Ag
S/Ag ratio
Killifish
LC_{50}
Embryo
NP Exposure in Killifish
AgNPs
polyvinylpyrrolidone (PVP)
Concentration

Figure S1. The full Instance Map of the ecotoxicity study. The full Instance map is on the previous page, which further depicts the Instance Map of figure 6 in the paper. The shortened map seen in figure 6 shows the material transformations that occur during the experimental study with the associated exposed organisms. The full Instance Map seen here contains the measured characterizations of the silver nanoparticles and aqueous media, as well as the measured endpoints of the exposed organisms categorized as properties across all phases of the study. Although the full map does not have the labeled phases as seen in figure 6, the phases directly correlate. When zoomed in, all of the measured characterizations and endpoints that were measured through the course of the study can be seen.
